# Sources and content of advice sought by parents/guardians prior to emergency department attendance

**DOI:** 10.1111/1742-6723.14514

**Published:** 2024-10-21

**Authors:** Scott McNeil, Nikita Goyal, Mandy Parr, John Cheek, Gary Freed, Alastair Meyer, Adam West, Simon Craig

**Affiliations:** ^1^ Monash Emergency Research Collaborative, Clinical Sciences at Monash Health Clayton Victoria Australia; ^2^ Department of Paediatrics, School of Clinical Sciences at Monash Health Monash University Melbourne Victoria Australia; ^3^ Paediatric Emergency Department, Emergency Program Monash Medical Centre Clayton Victoria Australia; ^4^ Emergency Department Royal Children's Hospital Melbourne Parkville Victoria Australia; ^5^ Clinical Sciences Murdoch Children's Research Institute Parkville Victoria Australia; ^6^ University of Melbourne School of Population and Global Health Melbourne Victoria Australia; ^7^ Emergency Program Casey Hospital, Monash Health Berwick Victoria Australia; ^8^ Faculty of Medicine, Nursing and Health Sciences Monash University Melbourne Victoria Australia

**Keywords:** general practitioner, paediatric emergency department, primary care, referral

## Abstract

**Aim:**

To describe sources of advice and the recommendations given to parents/guardians prior to attending ED with their child.

**Methods:**

This was a prospective observational study of patients presenting to two EDs of a multi‐centre Victorian Health service in June 2016. Data collection involved surveying all parents/guardians attending paediatric ED during a 1‐week period by trained research assistants. We determined the proportion of eligible respondents who sought advice before attending ED, the source of advice, and the type of advice provided.

**Results:**

One thousand sixty‐nine patients presented to ED over the 1‐week period. There were 730 responses to the survey, of which 65% (477/730) had received a total of 620 recommendations prior to ED attendance. Seventy‐six per cent (362/477) had received advice from a single source, 19% (90/477) had received advice from 2 sources, and 5% (25/477) from 3 or more sources. The most common sources of advice were general practice consultations (49%), friends/family (13.5%), and NURSE‐ON‐CALL (11%). Fifty‐four per cent (335/620) of the recommendations were to attend ED immediately and 12% (77/620) were to attend if their child was getting worse.

**Conclusions:**

Most parents and guardians sought advice from a single source prior to attending an ED. The most common source of advice was consultation with a general practitioner and the most common recommendation was to attend ED immediately, or if their child's condition worsened.

## Graphical abstract

Of 730 children attending 2 EDs over a 1‐week period, two‐thirds of parents/guardians obtained advice prior to attending ED, most commonly from general practitioners, telephone advice lines and specialists. The most common recommendations were to attend ED immediately, or if the child's condition worsens.

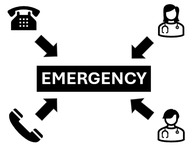

**Key findings**

- There has been an increase in the number of paediatric ED presentations and a decrease in paediatric primary‐care presentations.
- General practice (GP) referrals to paediatric EDs are increasing, especially for low‐acuity conditions.
- Less than half of parents/guardians consult a GP before attending ED.
- Approximately, two‐thirds of parents/guardians attending ED obtain advice before attending ED.
- Where parents seek advice, they are likely to obtain it from various health professionals such as GPs, NURSE‐ON‐CALL, and specialists.
- The most common recommendation made by all sources is to ‘attend ED immediately’ or ‘attend ED if worsening’.

## Introduction

In 2021–2022, there were over 8.8 million ED visits in Australia.^1^ Despite only making up 6% of the population, 10% of ED presentations were for children aged 0–4 years.^1^ The rate of paediatric ED attendance for this age group was significantly higher compared to other age groups at approximately 600 presentations per 1000 population compared to 339 for the general population.^2^

Increased patient volume in EDs can lead to delayed medical care, compromised quality of patient care, patient dissatisfaction, and increased risk of complications. While research suggests that neither a lack of access to primary‐care,^3^, ^4^ nor convenience significantly influences a patient's decision to attend ED, it remains a focus of national health policy.^4^

The increase in ED presentations among children has coincided with a decline in the overall proportion of General Practice (GP) visits made by children, and a decrease in the absolute number of extended GP consultations for children.^5^ These trends have emerged despite an overall increase in the paediatric population and the prevalence of chronic disease among children.^5^ This suggests a shifting pattern in healthcare‐seeking behaviour, with a greater reliance on ED service.

The exact reasons for this increase in paediatric presentations to ED are not fully understood but are thought to include a rise in GP referrals for low‐acuity conditions,^6^, ^7^, ^8^ parental appraisal of their child's illness as severe,^6^, ^8^ challenges in defining a primary‐care‐type patient^9^ and elevated expectations of paediatric care.^10^, ^11^

A 2009 study of low‐acuity patients (Australasian Triage Scale 4 or 5) children attending a single tertiary paediatric ED in Brisbane suggested deficiencies in primary care services, and a difference in perception between a ‘non‐urgent’ ED patient and one which cannot be dealt with in a primary care setting.^12^ Two‐thirds of children in this study had sought advice prior to ED presentation, while one quarter had not been able to access a GP.^12^

Although there is extensive research on referrals by GPs to paediatric EDs,^4^, ^5^, ^6^, ^7^, ^8^, ^10^, ^11^ there is little research to evaluate the overall sources and content of advice given prior to ED attendance. Understanding this can inform healthcare coordination and planning, enabling policymakers and healthcare institutions to make informed decisions regarding funding, public health campaigns, education programmes and resource allocation.

The primary objective of this paper is to describe sources of advice and the recommendations given to parents/guardians prior to attending ED with their child.

## Methods

### Study design

This was a prospective observational study that enrolled parents/guardians of children under 19 years of age who attended one of two EDs located in South‐East Melbourne. This study was approved in 2016 as a low‐risk study following a complete ethical review by the Monash Health Human Research Ethics Committee (16240Q).

### Setting

The EDs comprised a tertiary‐level paediatric ED (Monash Medical Centre) and a mixed urban district ED (Casey Hospital), both of which are located within the same hospital network, Monash Health. The tertiary ED had a yearly paediatric census of over 30 000, while the urban district ED had over 18 000 paediatric attendances per year. The data were collected over 1 week in June 2016 by trained research assistants (volunteer medical students) stationed in both EDs around the clock. It was estimated that there would be approximately 1000 paediatric presentations to ED during the week, which would serve as an adequate sample size for precise point‐estimates.

### Selection of participants

Parents/guardians of patients attending ED who were <19 years of age (an age cut‐off used to stream patients into ‘adult’ or ‘paediatric’ areas in the tertiary ED participating in the study) presenting during the 1‐week period were approached to be recruited. The study excluded patients who presented to ED without a parent or guardian; if the parent or guardian did not speak English; if they presented by ambulance or were transferred from another hospital; or if the treating clinician thought the survey was inappropriate, for example in cases such as critical illness.

Parents and guardians were provided with a verbal explanation of the nature of the survey prior to participation. Parents/guardians completed the questionnaire in the waiting room after being triaged. Where patients were immediately moved to a cubicle, the survey was administered after the patient was deemed stable by clinical staff. This was to ensure the questionnaire was properly administrated, minimise the risk of coercion and obtain accurate information. There was no follow‐up of the patients.

### Survey overview

Data were collected through a survey (Supporting Information Appendix S1) which contained specific questions relating to sources and content of advice prior to ED attendance. Respondents were able to indicate whether they had obtained advice before attending hospital, and the source of the advice (e.g. family member, friends, NURSE‐ON‐CALL^13^ or other telephone advice, general practitioner, specialist).

Additional information was requested regarding the content of advice; options included: ‘I wasn't given any advice’, ‘Go to the ED straight away’, ‘Go to ED if things get worse’, ‘Go to ED if things don't get better’, ‘See your GP’, and ‘Manage at home’. Free‐text boxes were available to record additional information regarding the source and content of advice. Where a response was not provided, this was specified as ‘not stated’.

Further questions (reported previously^9^) related to whether or not the parent/guardian felt that a GP would be able to look after the child's current illness/injury, and questions relating to various factors that may have influenced the decision to seek emergency care (such as proximity, previous experiences at the hospital).^9^ Following the completion of the questionnaire, the data were entered into a secure, purpose‐designed spreadsheet.

### Data collection and analysis

For analysis, sources of advice were combined into the following categories: ‘GP’, ‘GP (nurse/reception)’, ‘NURSE‐ON‐CALL’, ‘family/friends’, ‘hospital/specialist/locum’ and ‘other’. The three most common ‘other’ sources of advice were ‘school’, ‘previous ED visits’ and ‘ambulance’.

Data regarding ED triage category (using the Australasian Triage Scale [ATS] categories^14^), working diagnosis, management and disposition were collected from a retrospective review of the ED information system (Symphony, Version 2.31, Ascribe, Bolton, UK). This information was added to the data collected from the survey for each participant.

Descriptive statistics for categorical data are presented using number and percentage, while continuous data followed a non‐parametric distribution, and is presented using median and interquartile range. Comparisons between those who had and had not obtained advice before ED presentation were made using *χ* ^2^ tests for categorical data and Kruskall–Wallis tests for continuous data. Analysis was performed using SPSS for Windows (IBM SPSS Statistics for Windows, Version 24.0, Armonk, NY, IBM Corp, 2016).

## Results

**Figure 1:**
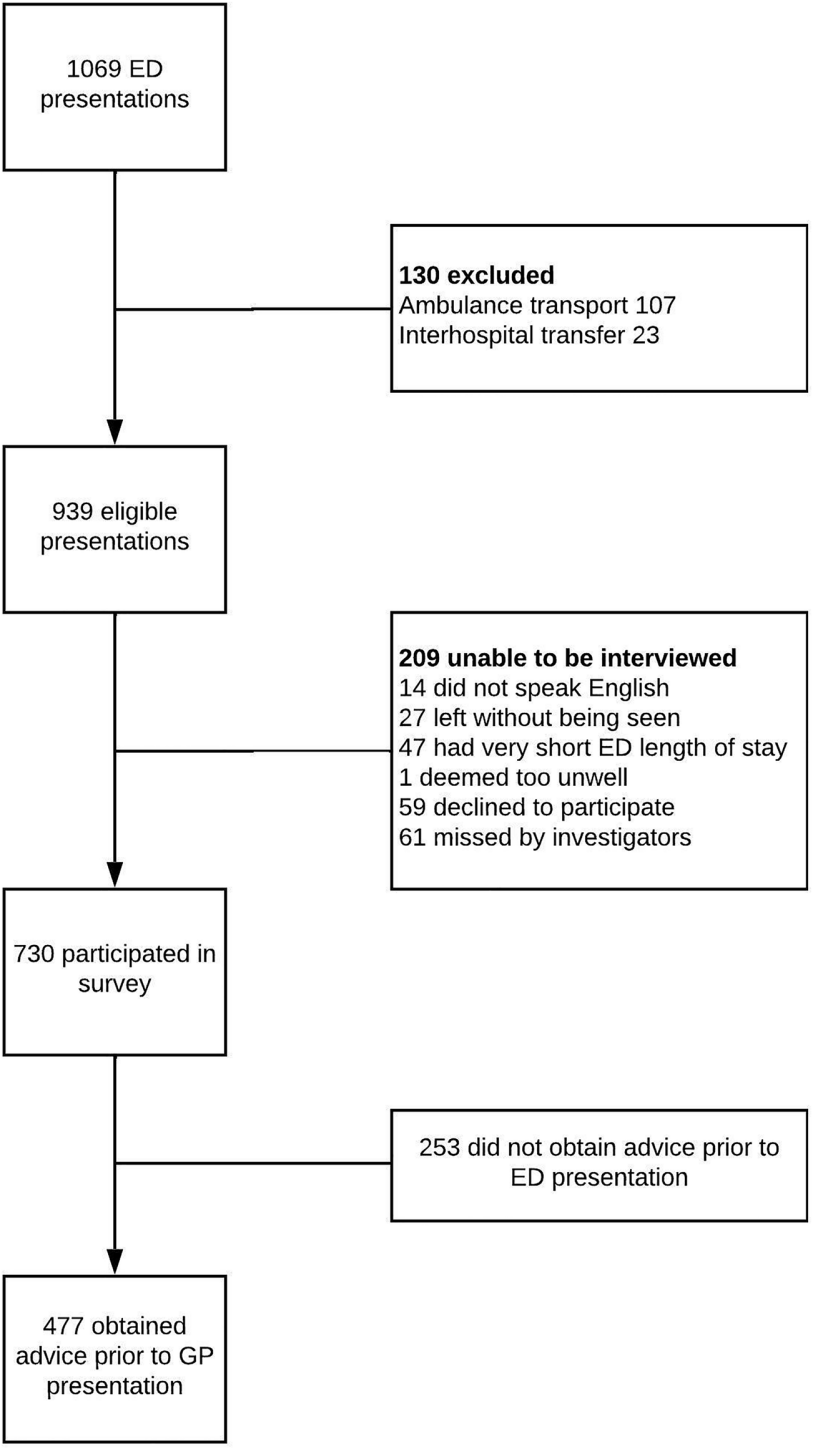
Recruitment diagram.

In the 1‐week data collection period in June 2016, 1069 patients presented to either the tertiary ED or the urban district mixed ED. One hundred thirty patients were excluded leaving 939 eligible presentations, of which 209 were unable to be interviewed. There were 730 responses to the survey, of which 65% (477/730) had received advice prior to attending ED (Fig. 1).

### Patient characteristics

The median age of a child included in the survey was 4 years old, with 55% (401/730) with a male predominance (Table 1). There was no significant difference in age, sex, hospital, or urgency (ATS Category 2) between those who obtained advice and those who did not (Table 1). However, those who did obtain advice were less likely to present after‐hours, had a greater ED length of stay, a longer median time between being seen by a clinician and discharged home, were more likely to be admitted or transferred to another hospital, and more likely to be categorised as ATS Category 3 than those who did not.

**TABLE 1:** Patient demographics

	Total number of parents/guardians who completed the survey (*n* = 730)	Parents/guardians who obtained advice (*n* = 477)	Parents/guardians who *did not* obtain advice (*n* = 253)	*P*‐value
Location/campus				
Tertiary Hospital (*n*, %)	420 (57.5)	271 (56.8)	149 (58.9)	0.59*
Presentation time				
After hours (between 6 pm and 7 am)	330 (45.2)	182 (38.2)	148 (58.5)	<0.001*
Median age (IQR)	4 (1–10)	4 (1–10)	4 (1–11)	0.12**
0–4 (*n*, %)	399 (54.7)	265 (55.6)	134 (53.0)	0.40*
5–9 (*n*, %)	134 (18.4)	85 (17.8)	49 (19.4)	
10–14 (*n*, %)	113 (15.5)	78 (16.4)	35 (13.8)	
15–18 (*n*, %)	84 (11.5)	49 (10.2)	35 (13.8)	
Sex				
Male (*n*, %)	401 (54.9)	263 (55.1%)	138 (54.5%)	0.88*
ATS category				
2 (*n*, %)	66 (9.0)	42 (5.8)	24 (9.5)	0.02*
3 (*n*, %)	204 (27.9)	150 (20.5)	54 (7.4)	
4 (*n*, %)	387 (53.0)	244 (33.4)	143 (56.5)	
5 (*n*, %)	73 (10.0)	41 (8.6)	32 (12.6)	
ATS Category 2	66 (9.0)	42 (5.8)	24 (9.5)	0.76*
Median length of stay, min (IQR)	170 (112.25–278.5)	182 (125–302)	155 (97–235)	<0.001**
Median time between being seen and being discharged, minutes (IQR)	89 (40–202)	100 (47–224)	65 (30–175)	<0.001**
Destination after ED visit				
Admit/transfer (*n*, %)	73 (10.0)	60 (12.6)	13 (5.1)	0.001*
Discharged/left without treatment (*n*, %)	494 (67.7)	304 (63.7)	190 (75.0)	
Short stay unit (*n*, %)	163 (22.3)	113 (23.7)	50 (19.8)	

*P*‐value calculated using *χ* ^2^ test (*) or Mann–Whitney U test (**) comparing those who obtained advice and those who did not. ATS, Australian Triage Scale.

### Source and content of advice

**Figure 2:**
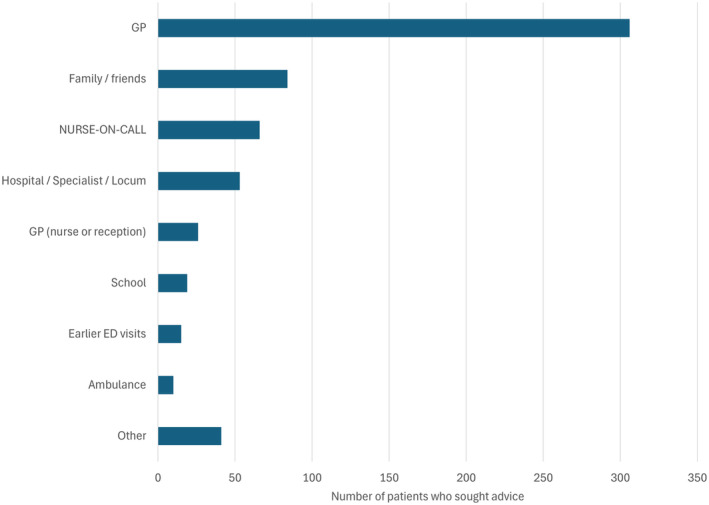
Number of patients who sought advice from each source. ‘Other’ includes advice from previous hospitalisation (eight), sports coach (eight), community nursing (seven), telephone advice line (five), locum service (three), Internet (three), first aid (three), other ED (one), ward nurse (one), pharmacy (one) and not stated (one). GP, general practitioner; ED, emergency department.

Overall, 477 respondents sought advice before ED attendance. A single source of advice was most common (76%, 362/477), while 19% (90/477) received advice from two sources and 5% (25/477) from three or more sources. The most common sources of advice comprised of consultations with general practitioners (49%, 306/620), family/friends (13.5%, 84/620) and NURSE‐ON‐CALL (11%, 66/620) (Fig. 2). Of the 306 consultations with GP, 71% (217/306) of these were the usual GP, while 21% (63/306) were with another GP (not with the family's regular GP), and the remaining 8% (26/306) were over the telephone or a GP home visit.

**Figure 3:**
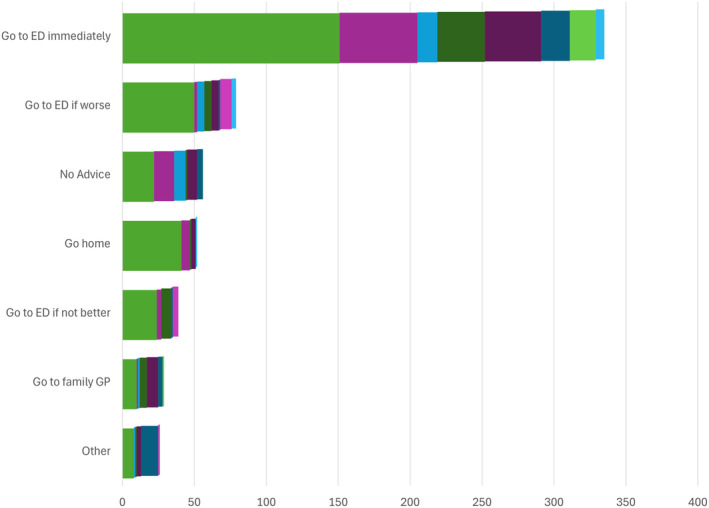
Number of recommendations by type and the source of advice. ‘Other’ includes advice from previous hospitalisation (eight), sports coach (eight), community nursing (seven), telephone advice line (five), locum service (three), Internet (three), first aid (three), other ED (one), ward nurse (one), pharmacy (one) and not stated (one). GP, general practitioner. 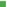, GP; 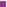, family/friends; 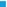, GP (nurse/reception); 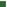, hospital/specialist/locum; 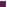, NURSE‐ON‐CALL; 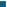, other; 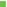, school; 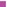, earlier ED visits; 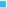, ambulance.

A total of 620 recommendations were obtained, with at least two recommendations provided to nearly one quarter of parents/guardians. The most common recommendation was to attend the ED immediately (54%, 335/620) or to attend the ED if the child was getting worse (12%, 77/620) (Fig. 3).

Of those receiving advice, 277/477 (58%) were advised to attend ED immediately by at least one source. Those advised to attend ED immediately were more likely to be admitted or transferred to another hospital (odds ratio 1.95; 95% CI 1.1–3.4; *P* = 0.02), however, were not more likely to receive any investigations (odds ratio 1.35; 95% CI 0.93–1.97; *P* = 0.12).

## Discussion

Approximately two‐thirds of parents/guardians who responded to the survey sought advice prior to attending the ED with their child. The most common source of advice was a general practitioner, while other prominent sources included friends/family, and telehealth (either GP – phone, NURSE‐ON‐CALL). The most frequent advice given was to attend the ED straight away, or to attend the ED if the child's condition worsened.

Referral to the ED by a GP is shaped by clinical assessment of illness severity and/or urgency, any direct referral request from the parent/caregiver, the GP's appraisal of parental coping ability, and timely availability of diagnostic tests and specialist care.^8^ There has been an increase of 60% in paediatric referrals by GPs to EDs between 2005 and 2015,^7^ especially for low‐urgency conditions,^6^, ^7^ and close to half of low‐urgency presentations to ED are paediatric.^15^ The increase in referral is likely complex, with potential reasons including limited after‐hours access, high parental expectations for specialist referral,^10^ rapid diagnosis and/or treatment,^10^, ^11^ and limited reimbursement which has been exacerbated in recent years due to a failure of the Medicare rebate to keep pace with inflation.^16^

The proportion of parents/guardians in our study who obtained advice from a GP (42.5%, 306/730) prior to attending ED was higher than previously reported in Victoria in 2016 (28%),^6^, ^7^ Queensland (35%)^12^ and South Australia (19.6%).^17^ The first two studies only included paediatric patients which were triaged as having lower urgency (ATS Category 4 or 5) presentations, although the discriminatory value of the ATS for urgency of non‐emergent paediatric presentations has been questioned.^18^ The South Australian study, focused on all patients attending ED and only 5% of the sample was less than 18 years of age.

In Victoria, it was also found that one‐fifth of parents/guardians contacted a nurse telephone triage service,^6^ almost double the amount (11%) that contacted NURSE‐ON‐CALL in our study. Other sources of advice in prior research include specialist advice,^12^ telephone to the ED prior to attending^12^ or contacting the National Healthdirect Australia triage hotline.^17^

While the specific content of advice has not been investigated in Australia, the frequency of which patients that are instructed to attend ED has previously been determined. A Victorian study of low‐urgency paediatric presentations found that two‐thirds were instructed to go to ED by their GPs^6^ and 70% were told to go to ED by the nurse telephone triage service.^6^ Likewise, in Queensland, almost half of low‐urgency paediatric patients who sought advice from various sources (including the GP and paediatrician) were advised to go to ED.^12^ These findings were consistent in our study where patients were most frequently advised to ‘attend ED immediately’ by both GPs and NURSE‐ON‐CALL.

These trends have also been present overseas. In the United Kingdom, a study of paediatric patients that were triaged as ‘low urgency’ (defined as patients triaged P3‐P6 on a modified Manchester Triage Scale^19^) found that 60% attempted to contact a GP^19^ and 60% of those seen by the GP were advised to attend ED.^19^ Other common sources of advice in the United Kingdom were found to be the Minor Injuries Unit, National Health Service Direct and schools.^19^ A study of patients of all ages in the United States that presented to the ED, a significantly lower proportion sought advice from a GP (38%),^4^ but nine times out of 10, they were told to go to ED.^4^

Utilising ATS categories as a surrogate marker for ‘ambulatory’ or ‘GP type’ is unreliable.^20^ General practitioners recommend patients who fall into these ‘low‐urgency’ categories to attend ED as they require further monitoring, investigation or treatment that are not readily available in clinics or after‐hours settings. GP referral to ED is often appropriate and more indicative of more severe illness and greater resource utilisation than self‐referral.^21^, ^22^ Improving resource allocation for primary care and education could provide the support needed for general practitioners to manage these lower‐acuity conditions on their own.^7^

Limited GP availability (during business hours)^3^, ^20^ and financial barriers do not appear to contribute to increasing paediatric ED presentations in those that do not obtain advice prior to ED attendance.^3^ Instead, for this group, factors such as the consideration of the severity and duration of a child's illness,^6^ alongside perceived benefits (more resources and higher efficiency)^11^ of ED and low parental confidence in GPs^23^ may be drivers of increasing ED presentations. Difficulties parents/guardians may face in assessing the urgency of their child's condition^19^ may be addressed by implementing educational interventions.^19^

There are several limitations of this study. The data were collected over a 1‐week period in June 2016 from only two urban EDs (one tertiary, one non‐tertiary). Hence, the cross‐sectional data may not adequately encapsulate the changes in motivators for ED attendance that may occur over an extended period. Furthermore, these data were collected prior to the COVID‐19 pandemic when telehealth, urgent care centres, virtual EDs and other diversion strategies were less prevalent than now.^24^ In addition, accessibility of general practitioner appointments, and use of social media and other internet searches for advice may have changed in the several years since this study took place. Accordingly, these data may not reflect the current pattern of referrals to paediatric EDs.

While efforts were made to ensure that all paediatric ED presentations were a part of the data set by ensuring that trained research assistants were present at all times of day, just over a quarter of these presentations were unable to be included. Reasons included the child being deemed too unwell to approach, declining to participate, or leaving the ED prior to being seen. It is possible that there is sampling bias and exclusion bias which may have resulted in an inaccurate representation of all ED presentations during that week, thus further limiting the generalisability of our results.

Given the changes to health‐seeking behaviour and increasing use of telehealth after the COVID‐19 pandemic, we believe further research is needed. To address the limitations outlined above, future studies could be conducted at a larger number of hospitals, with intermittent sampling over a longer period, and/or using electronic surveys (e.g. SMS or email) to parents/guardians after their ED attendance. Further research could also explore the outcomes of those advised to attend ED immediately.

## Conclusion

In this study, two‐thirds of parents/guardians obtain advice prior to attending ED. When seeking advice, most parents/guardians turn to a range of healthcare professionals, including general practitioners, NURSE‐ON‐CALL services, and specialists. The most common recommendation was to attend ED immediately or if child's condition worsens.

## Supporting information


**Appendix S1.** Supporting Information.

## Data Availability

The data that support the findings of this study are available from the corresponding author upon reasonable request.
