# Supplementary material for: Sources and content of advice sought by parents/guardians prior to emergency department attendance
Source: Emerg Med Australas. 2024 Oct 21;37(1):e14514. doi: 10.1111/1742-6723.14514 (PMC11744407; doi:10.1111/1742-6723.14514)
Supplement: Supplementary file 1 — Appendix S1. Supporting Information. [file EMM-37-0-s001.pdf]

1. What is the patient's UR number?

[ ]

2. What is the date and time of presentation (from Symphony screen)?
NOTE- the program will not accept 24h clock notation (e.g. 00:40, 15:00). Please use **12-hour clock time**

	DD	MM	YYYY	hh	mm	AM/PM
Date / Time	[ ] /	[ ] /	[ ]	[ ] :	[ ]	-

3. Was the patient transferred from another hospital?

- ○ Yes
- ○ No

4. Was the patient brought to hospital by ambulance?

- ○ Yes
- ○ No

5. Has the patient / family refused consent?

- ○ No
- ○ Yes

6. Does the patient / family speak English well enough to complete the survey?

- ○ Yes
- ○ No

7. What is YOUR name?

[ ]

8. Who brought the patient to the emergency department today?
(please select ALL that apply)

- [ ] Mother
- [ ] Father
- [ ] Sibling
- [ ] Grandparent
- [ ] Other (please specify)

9. Who is completing this survey?
(please select ALL that apply)

- ○ The person accompanying the patient / child
- ○ The patient / child
- ○ Both

w process

Question 3 answers

10. Q3 Answer grid

Please provide answers to the "advice given" columns ONLY in this question

	I wasn't given any advice	Go to the ED **straight away**	Go to the ED **if things get worse**	Go to the ED **if things don't get better**	See my GP	Manage at home
Family member	☐	☐	☐	☐	☐	☐
Friends	☐	☐	☐	☐	☐	☐
Telephone to "Nurse on Call"	☐	☐	☐	☐	☐	☐
Telephone to usual GP - spoke to **reception staff**	☐	☐	☐	☐	☐	☐
Telephone to usual GP - spoke to **nursing staff**	☐	☐	☐	☐	☐	☐
Telephone to usual GP - **spoke to GP**	☐	☐	☐	☐	☐	☐
Visited my **usual GP**	☐	☐	☐	☐	☐	☐
Visited **another GP** (not my usual doctor)	☐	☐	☐	☐	☐	☐
Visited **at home** by my **usual GP**	☐	☐	☐	☐	☐	☐
Visited at home by a locum (home-visiting) doctor	☐	☐	☐	☐	☐	☐
Telephone to hospital	☐	☐	☐	☐	☐	☐
Telephone to usual specialist	☐	☐	☐	☐	☐	☐

Other advice provided - please describe source of advice, and advice given.

ONLY do this for the bottom row of Q3 (i.e. another source of advice)

11. Q3 Answer grid

Please provide answers to the "other advice" column

Family member

Friends

Telephone to "Nurse on call"

Telephone to usual GP - spoke to **reception staff**

Telephone to usual GP - spoke to **nursing staff**

Telephone to usual GP - **spoke to GP**

Visited my **usual GP**

Visited **another GP** (not my usual doctor)

Visited **at home** by my **usual GP**

Visited at home by a locum (home-visiting) doctor

Telephone to hospital

Telephone to usual specialist

12. Question 4

	Strongly disagree	Disagree	Agree	Strongly agree
My child's illness / injury is serious				
My child's illness has been going on for too long				
My child's fever is not going away				
I like the idea that if my child needs x-rays or blood tests they can be done more easily in the emergency department				
I couldn't get off work to bring my child to the GP during regular office hours				
It is more convenient for me to bring my child to the emergency department than to the GP				
I never have to pay for care at the emergency department				
The hospital is closer to my home than the GP				
I think a GP would be able to look after my child's current illness / injury				

13. Question 5

	Not at all important	Not very important	Important	Very important	N/A
My child has a chronic illness managed by Monash Medical Centre / Monash Children's	○	○	○	○	○
I trust Monash Medical Centre to take care of my child	○	○	○	○	○
I don't trust any other hospital to take care of my child	○	○	○	○	○
Other hospitals do not take my concerns seriously	○	○	○	○	○
Monash Medical Centre was the closest emergency department to my home	○	○	○	○	○
My GP referred me here	○	○	○	○	○
My paediatrician referred me here	○	○	○	○	○
I have come to Monash Medical Centre emergency in the past and liked it	○	○	○	○	○
I want the doctor in the emergency department to speak English very well	○	○	○	○	○
I thought my child needed to see a specialist for the problem today	○	○	○	○	○

14. Thank you

You have completed data entry for this patient.

The survey will now allow you to enter data for another patient's presentation

Under review process
